# Postoperative use of drain in thyroid lobectomy – a randomized clinical trial conducted at Civil Hospital, Karachi, Pakistan

**DOI:** 10.1186/1756-6614-5-9

**Published:** 2012-09-28

**Authors:** Zahid Ali Memon, Gulrayz Ahmed, Sarah Rafi Khan, Mahvesh Khalid, Naheed Sultan

**Affiliations:** 1 Senior Registrar, Surgical Unit 1, Civil Hospital, Karachi, Pakistan; 2 4th Year Student, Dow Medical College, DUHS, D – 31, Block 8, Gulshan-e-Iqbal, P.O Box 75300, Karachi, Pakistan; 3 Dean Surgery and Allied, Incharge Surgical Unit 1, Dow University of Health Sciences, Civil Hospital, Karachi, Pakistan

**Keywords:** Thyroidectomy, Lobectomy, Drain, Postoperative, Complications

## Abstract

**Background:**

Thyroidectomy is a common surgical procedure, after which drains are placed routinely. This study aims to assess the benefits of placing postoperative drains, its complications and affects on postoperative stay, in thyroid lobectomy.

**Methodology:**

Randomized Clinical Trial of 60 goitre patients undergoing lobectomy was conducted at Civil Hospital Karachi, during July’11-December’11. Patients were randomly assigned into drain and non drain groups. Patient demographics, labs and complications were noted. Ultrasound of neck was performed on both groups. For drain group, the amount of fluid present in the surgical bed and redivac drain was added to calculate fluid collection while in non drain group it was calculated by ultrasound of neck on first and second post-op days. Data was entered and analyzed on SPSS v16 using Independent T tests.

**Result:**

The mean total drain output for 2 days in non-drain group was significantly lower 10.67 (±9.072) ml while in drain group was 30.97 (±42.812) ml (p = 0.014). The mean postoperative stay of drain group (79.2 ±15.63 hours) was significantly higher, as compared to mean postoperative stay of non drain group (50.4 ±7.32 hours). Mean Visual Analogue Score (VAS) for pain day 1 (6.2 ±0.997) and day 2 (4.17 ±0.95) in drain group were significantly higher compared to day 1 (2.6 ±1.163) and day 2 (1.3 ±0.877 ) of non drain group. From drain group, 2 patients complained of stridor, dyspnea on Day 1 which subsided by Day 2 and 1 case of voice change, with no such complains in non drain group. No patients from both groups developed seroma, wound infection or hematoma.

**Conclusion:**

In uncomplicated surgeries especially for lobectomy, use of drain can be omitted.

## Introduction

Thyroidectomy is one of the most commonly performed operative procedure in general surgery [1]. Indications for thyroid surgery are hyperthyroidism, thyroid swellings, and thyroid cancers. Indications for thyroid surgery are hyperthyroidism, thyroid swellings, with a prevalence of between 4.2 to 51.3% [2], and thyroid cancers.

After thyroid surgery, the chief reason for surgeons placing a drain is to detect early postoperative hemorrhage [3], and to avoid its risk of blocking the respiratory passage [4]. However, a common problem is that the drains become blocked with clotted blood and are useless in alerting the surgeon even if major bleeding occurs [1]. The probability of a postoperative hematoma forming after thyroid surgery ranges between 0 to 30% [5]. However, past studies have failed to show that placement of drains prevent the hematoma formation.

There are also very low chances of postoperative seromas forming in the absence of drains but they can be observed and allowed to resorb themselves or, if severe, aspirated [6].

Past studies conducted on the usefulness of drain placement after thyroid surgery have failed to show any benefits [7]. Instead, it was found that usage of drains increased the chances of surgical wound infections [8]. From two studies conducted in Pakistan, both reported that the use of drains is not mandatory after thyroidectomy provided that strict principles of haemostasis are followed [6,9]. The study done in 2005 showed no difference in the prevalence of post thyroidectomy hematoma formation whether drains were used or not [6]. The other study reported greater fluid collection and more postoperative complications in the group of patients with drains than those without [9]. There have not been, however, many recent studies in this part of the world showing whether the use of drains is mandatory or not.

In our clinical setup, drains are commonly placed postoperatively. We have conducted this trial to see if results from other studies are relevant to ours and if disadvantages of the use of drains significantly outweigh its advantages. The use of drains after thyroid surgery is being questioned worldwide now that surgical techniques have improved for thyroid disorders. This study aims to assess the necessity of drains and to eliminate their routine use after thyroid surgery. Also, the aim for this study is to compare our findings with those of international studies and also for general awareness.

## Materials and methods

A randomized clinical trial of diagnosed patients of goiter was conducted at Civil Hospital, Karachi between July 2011 and December 2011. All the patients who were to undergo sub-total lobectomy or lobectomy during the study period, were selected and randomized in to two equal groups, one with drain placed post-operatively and the other without drain. Allocation of patients to drain and non-drain group was done on the basis of computer generated random number table. Just before wound closure, the operating surgeon was informed of the group. Cases of total thyroidectomy, subtotal thyroidectomy, thyroid carcinomas or previous thyroid surgery were excluded. Cases of goiter were confirmed by all or atleast two of the following tests; ultrasound (U/S) neck, fine needle aspiration for cytology (FNAC), thyroid scan and histopathology. Indirect and direct laryngoscopy was performed preoperatively and postoperatively as a routine protocol.

The study instrument was a structured questionnaire that was filled by medical students after obtaining informed consent from the patients. The questionnaire was divided into four parts. The first part included demographics of the patient. The second part included baseline labs, including hemoglobin levels, platelet counts, total leucocytes counts, blood urea nitrogen, creatinine, serum thyroid stimulating hormone, T3 and T4 levels. The third part had details pertaining to the tests performed to confirm diagnosis of goiter. Postoperative complications were noted down in the fourth part. The patients were inquired about complications including pain, stridor, dyspnea and voice change. Pain and discomfort were evaluated by VAS (Visual Analogue Scale) with 0 being no pain and 10 being severe unbearable pain. Complications like wound infection, seroma, hemorrhage and hematoma were also noted.

Drain output was noted on post operative day 1 and day 2. In the drain group, a negative pressure drain was placed postoperatively. Ultra sound of the neck was performed in both groups, using B mode with linear frequency of 7.5 MHz. It was performed by the same radiologist with fluid volume being calculated by measuring the maximum diameter of the operative bed in three dimensions. For the drain group, the amount of fluid present in the surgical bed and Redivac drain was separately calculated and added to calculate fluid collection while in the non drain group it was calculated by ultrasound of neck. Then the calculated amount of fluid for both groups was compared. Total drain output was calculated by adding fluid collection of both groups for the two days.

Data was entered and analyzed on SPSSv16. Differences between both groups of age, postoperative hours of stay, Pain Day 1 (PD1), Pain Day 2 (PD2), Drain Output Day 1, Drain Output Day 2 and Total Drain output for two days (TD) variables was analyzed using Independent T tests with p values less than 0.05 being significant. Mean VAS scores for Pain Day 1 (PD1) and Pain Day 2 (PD2) were also analyzed using Independent T Test. Simple descriptive analysis was used to calculate frequencies, percentages and means of all variables. Results were represented in pie charts, bar charts, tables and cross tabs.

### Ethical consideration

This trial was approved by the concerned Surgical Unit Ethical Committee. Written Consent was obtained from the participants after explaining the study objectives. The participants were free at all times, to withdraw from the trial without giving any reason. Strict measures for confidentiality were maintained throughout the process of data collection, entry and analysis. All efforts were made in this study to fulfill the ethical considerations in accordance with the ‘Ethical principles for medical research involving human subjects’ of Helsinki Declaration [10].

## Result

**Figure 1:**
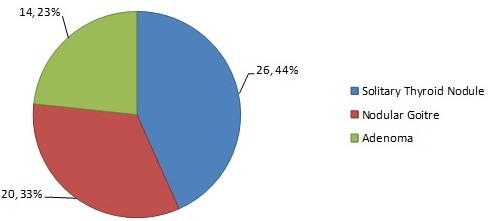
Total Diagnosed Cases.

Of the 60 patients, 6 (10%) were males and 54 (90%) were females with a ratio of 1:9. Drain group included 3 males and 27 females with mean age of 32.27 (±9.663) years, of which 16 were, diagnosed solitary thyroid nodule, 7 had nodular goitre and 7 cases of adenoma. Non drain group had 3 males and 27 females with mean age of 31.23 (±7.596) years, of which 10 were diagnosed cases of solitary thyroid nodule, 13 nodular goitre and 7 adenoma. Diagnosis of all cases is represented in Figure 1.

The mean calculated TD in non drain group (10.67 ±9.072 ml) was significantly reduced as compared to drain group (30.97 ±42.812 ml, IQR = 18.25 ml, Median = 25 ml) (p = 0.014). The relationship between fluid collection of both groups on Day 1 and Day 2 is represented in Table 1.

**Table 1:** Drain Output Day 1 and 2

	**No Drain**				**Total**	**p-value**	**Drain**				**Total**	**p-value**
	**0 – 09 ml**	**10 – 19 ml**	**20 – 29 ml**	**> 30 ml**			**0 – 09 ml**	**10 – 19 ml**	**20 – 29 ml**	**> 30 ml**		
**Day 1**	11	17	2	0	30`		8	11	8	3	30	
**%**	36.67	56.67	6.67	0	100	0.228	26.67	36.67	26.67	10	100	0.61
**Day 2**	22	8	0	0	30		11	12	3	4	30	
**%**	73.3	26.7	0	0	100		36.67	40	10	13.33	100	

**Figure 2:**
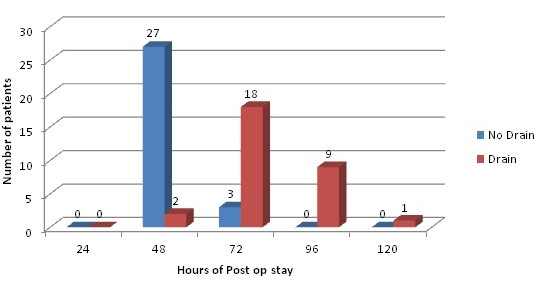
Post-op stay.

The mean post-op stay of drain group was 79.2 (±15.63) hours which was significantly higher, as compared to mean post op stay of 50.4 (±7.323) hours in non drain group (p < 0.001). Details of post-op stay are mentioned in Table 2 and compared diagrammatically in Figure 2.

**Table 2:** Post-Op Stay

**Group**	**Hours**					**Total**	**P value**
	**24**	**48**	**72**	**96**	**120**		
**No Drain**	0	27	3	0	0	30	
**%**	0	90	10	0	0	100	<0.001
**Drain**	0	2	18	9	1	30		
**%**	0	6.7	60	30	3.3	100		

The mean VAS score also showed significance. In drain group mean VAS score for PD1 was 6.2 (±0.997) and for non drain group 2.6 (±1.163) (p < 0.001). For PD2 mean VAS score was also significantly higher in drain group being 4.17 (±0.95) as compared to 1.3 (±0.877) for the non drain group (p < 0.001).

From drain group (n = 30), 2 (6.67%) patients complained of stridor and dyspnea (respiratory complications) which subsided by Day 2, and 5 (16.7%) cases complained of voice change on Day 1, of which 1 had the same complain next day. There was no complaining of stridor, dyspnea or voice change in non drain group. Complication of hemorrhage (200 ml) was noted in a patient of drain group who was re-operated for haemostasis. None of the patients in both groups developed hematoma, wound infection or seroma. Table 3 compares the number of complications in both groups.

**Table 3:** **Comparison of complications in  both groups**

			**Drain (n = 60)**		**P value**
			**No (n = 30)**	**Yes (n = 30)**	
Respiratory Complications Day 1	No	Count	30	28	0.150
		% within Drain No / Yes	100%	93.3%	
	Yes	Count	0	2	
		% within Drain No / Yes	0%	6.7%	
Respiratory Complications Day 2	No	Count	30	30	-
		% within Drain No / Yes	100%	100%	
	Yes	Count	0	0	
		% within Drain No / Yes	0%	0%	
Voice Change Day 1	No	Count	30	25	0.020
		% within Drain No / Yes	100%	83.3%	
	Yes	Count	0	5	
		% within Drain No / Yes	0%	16.7%	
Voice Change Day 2	No	Count	30	29	0.313
		% within Drain No / Yes	100%	96.7%	
	Yes	Count	0	1	
		% within Drain No / Yes	0%	3.3%	
Hemorrhage	No	Count	30	29	0.313
		% within Drain No / Yes	100%	96.7%	
	Yes	Count	0	1	
		% within Drain No / Yes	0%	3.3%	

No complications of Hypocalcemia, Wound Infection and Seroma reported.

## Discussion

Lobectomy or subtotal thyroidectomy is one of the most common surgical procedures. It is usually performed for benign thyroid disorders like multinodular goitre, nodular goiter, and adenomas.

It is common practice for surgeons to routinely insert a drain after every case of thyroid surgery [4], whether it is total thyroidectomy or lobectomy. This is mainly due to the fear of postoperative hemorrhage [3] or accumulation of excess lymphatic fluid which needs to be drained as it can compromise the airway [4]. Postoperative bleeding after thyroid surgery is reported to be as rare as 0.3 to 1% [1], while the probability of a postoperative cervical hematoma forming ranges between 0.1 to 4.7% [11]. In two studies of 250 and 400 patients no benefit of using drains after thyroid surgery has been documented [12,13]. It has been observed that if correct surgical techniques and hemostatic procedures are followed, excessive post-operative bleeding can be avoided, decreasing the incidence of hematoma formation. Precautions such as staying within the subplatysmal plane during surgery and using coagulation diathermy along with proper ligation of bleeding vessels will reduce chances of postoperative hemorrhage [6].

In real practice insertion of drain should be rationalized on the basis of the operative procedure performed and the extent of neck dissection along with patient to patient variation. Many authors recommend the use of drains only for complicated cases such as resection of substernal goiter, large dead space, raw thyroid bed [13,14] or in hypervascular diseases of thyroid (e.g. Grave’s disease) or certain carcinomas [15]. In our study, only one patient with drain developed complication of hemorrhage and required reoperation in which the amount of fluid was 200 ml. Thus prevalence of this complication was 3.33% in the drain group of this study which is comparable to international figures showing a variable prevalence of between 0.1 to 4.7% [11]. A study from Hyderabad, Pakistan reported 1.4% prevalence of this complication which is comparatively lower than this study [16].

In the present study, there were no cases of seroma formation in either the drain or non drain group, which coincides with the fact that seroma formation does not specifically occur when drains are not used. No patients in any group had to undergo aspirations (except for a case hemorrhage). Conversely, the placement of drains itself can lead to seroma formation since the drain is a foreign object. A study showed that there was fluid collection in the surgical field regardless of the use of a drain, the reason being, either the drain triggered inflammation and fluid formation itself or the negative pressure created by the drain sealed off the lymphatics [2]. Likewise, in this study the amount of fluid in the drain group was significantly greater [30.97 (±42.812) ml] than the non-drain group [10.67 (±9.072) ml] (p < 0.001). So placement of drains can initiate or even further aggravate an already developing seroma.

The insertion of drain after every thyroid surgery increases the risk of introducing infection into the patient. When compared with international literature, the incidence of wound infections after thyroid surgery in the drain group showed variable results, from no wound infections in some studies to up to 3.4% in others [1,7]. Hence, post surgical wound management and aseptic techniques used during surgery also play a major role in determining the development of infection.

Similarly, our study also suggests that insertion of drain after the thyroid surgery increases the hospital stay of the patients [79.2 (±15.63) hours] as compared to the ones that were left without the drain [50.4 (±7.323) hours]. In a study done in 2010, it was found that the length of hospital stay was increased by a day in these patients, which obviously increased overall costs [2]. Another study documents that use of drain in uncomplicated thyroid surgeries, not only increases the duration of hospital stay but also the chances of infection [17]. Hence, this also increased the cost beared by the hospital. This would not benefit a hospital setting, as in our study, which caters to an ever-growing number of patients free of cost with limited number of beds.

Patient discomfort and pain were assessed on day 1 and 2 after surgery, via the VAS (Visual Analogue Scale). It showed that the patients in the drain group felt a greater degree of pain and discomfort than those in non-drain group. The mean VAS score for PD1 and PD2 in drain group was significantly higher (p <0.01) as compared to non drain group. This consequently led to a greater use of analgesics. Our results coincided with those of an African study done in 2011, which also showed the mean VAS to be significantly reduced in the non-drain group on postoperative day 0 and 1 [7]. Hematoma normally becomes clinically apparent after 4 to 6 hours of surgery [18], but no such case was recorded.

Placement of drains can also cause poor cosmetic results and create separate surgical scars. It also increases operating time by 5 minutes due to the need for an extra, irregularly shaped stab wound to be made in the lower neck or chest [6]. Other complications can include hypoparathyroidism and hypocalcemia, voice change, stridor, and dyspnea; of which there were no cases, besides one case of voice change in drain group, in our study.

The present clinical trial, in conformity with numerous international clinical trials [1,3-5,7], could not show any benefit of routinely placing drains after every case of thyroid surgery, particularly uncomplicated cases. Albeit, it did display the complications as well as life threatening conditions which can possibly occur if drains are placed routinely. This study demonstrated that prevention of postoperative hematoma did not require drain placement as much as performance of correct hemostatic techniques were needed and also that seroma formation can be triggered by drains themselves. It also showed that postoperative stay, costs, patient discomfort and pain are greater in patients which have drain placement after thyroid surgery. Major limitations of the study included patient’s understanding to questions asked, unequal distribution of benign diagnosed goiter cases in both groups and chances of errors while estimating fluid volume.

## Conclusion

In uncomplicated surgeries, especially in cases of lobectomy; use of drain can be omitted which will help decreasing chances of wound infection, with substantial shortening of patient stay while increasing patient comfort and satisfaction.

## Abbreviations

VAS: Visual Analogue Scale; TD: Total Drain output for two days; PD1: Pain Day 1; PD2: Pain Day 2.

## Competing interests

The authors declare that they have no competing interests.

## Authors’ contributions

ZAM and PN were involved in conceptualizing the study and final paper reviewing. GA was also involved in the conceptualization of this study, questionnaire development and wrote the initial and final paper and performed data analysis with assistance from SRK. MK supervised the data collection, entered and verified the data and helped in drafting the initial paper. All authors read and approved the final manuscript.
